# Qualitative and Quantitative Ovarian and Peripheral Blood Mitochondrial DNA (mtDNA) Alterations: Mechanisms and Implications for Female Fertility

**DOI:** 10.3390/antiox10010055

**Published:** 2021-01-05

**Authors:** Andrea Busnelli, Annalisa Navarra, Paolo Emanuele Levi-Setti

**Affiliations:** 1 Department of Biomedical Sciences, Humanitas University, Pieve Emanuele, 20090 Milan, Italy; 2 Humanitas Clinical and Research Center, IRCCS, Department of Gynecology, Division of Gynecology and Reproductive Medicine, Fertility Center, Rozzano, 20089 Milan, Italy; annanavarra@hotmail.it (A.N.); paolo.levi_setti@humanitas.it (P.E.L.-S.)

**Keywords:** mitochondrial DNA, mtDNA, ovarian reserve, fertility, subfertility, oxidative stress, mtDNA copy number

## Abstract

The reduction of female fertility over time is considered as a natural consequence of ovarian aging. The exact mechanism underlying this process is not fully elucidated. However, it is becoming increasingly evident that qualitative and quantitative mitochondrial genome alterations might play a relevant role. The former include mitochondrial DNA (mtDNA) damage caused by oxidative stress, the accumulation of acquired mtDNA mutations, the effects of inherited mtDNA mutations, and alterations in the mitochondrial stress response mechanism. The latter refer to alterations in the oocytes, granuolosa cells, and embryonic cells mtDNA content. The present review aims to investigate the evidence about: (1) the effect of qualitative and quantitative mtDNA alterations on female fertility, paying particular attention to those with a pathophysiology characterized by a relevant role of oxidative stress; (2) the use of oocytes, granulosa cells (GCs), embryonic cells, and peripheral blood cells mtDNA copy number as a female fertility surrogate biomarker; (3) experimental therapies tested to try to subvert the ovarian aging process with particular reference to antioxidant treatments.

## 1. Introduction

Women attain the peak of fertility in their early and mid-20s. After that, fecundity starts to decline gradually. This decrease is initially slight and, after 35 years of age, becomes more pronounced [1]. The reduction of female fertility over time is considered the natural consequence of ovarian aging. This process includes both quantitative and qualitative alterations of the ovarian reserve [2]. Older oocytes development is characterized by a high incidence of meiotic errors and, as a result, of aneuploidies and alterations of cells’ organelles. The reproductive competence of such oocytes is thus often impaired [1]. Epidemiological data from the last decades demonstrate a progressive increase of age at which women bore their first child. Considering the detrimental effect of aging on fertility, the observed trend is worrying. Not surprisingly, studies aimed at forecasting future population characteristics showed that the steadily increasing education level on the one hand and contraceptives demand on the other will lead to a progressive decline in fertility and in population growth all around the world [3].

The exact mechanism underlying the ovarian aging process is not fully elucidated. However, it is becoming increasingly evident that mitochondrial genome alterations can play a relevant role [4].

Mitochondrial DNA (mtDNA) is a double-stranded, circular 16,568 base pairs DNA molecule encoding 37 genes. Mitochondrial ribosomal and transfer RNA cooperate for the synthesis of 13 peptides. Noteworthy, mitochondrial genome, due to the lack of protective histones, is particularly susceptible to the oxygen free radicals attack and to somatic mutations development. An excess in mtDNA mutation may determine the alteration of the oxidative phosphorylation (OXPHOS) process and, as a result, the onset of diseases associated with mitochondrial dysfunction [4,5].

The mature human oocyte is the human cell with the highest mitochondria and mtDNA content. This is probably due to the considerable energy required for fertilization and early embryonic development. Oocyte mitochondrial replication begins during fetal life: oogonia initially contain approximately 200 mitochondria. This number progressively rises as oocytes proceed through their process of replication and maturation. Notably, an oocyte at metaphase II (MII) stage contains roughly 100,000 mitochondria and between 50,000 and 550,000 mtDNA copies depending on the species and on the assessment technique used [6,7,8,9]. Importantly, the mtDNA replication capacity is not reactivated until the embryo reaches the blastocyst stage. As a consequence, the embryo expansion is characterized by a dilution in the number of mitochondria. Not surprisingly, one may observe a reduced mtDNA content in blastocyst stage embryonic cells when compared to that in oocytes. It follows that, in the period of time elapsing from fertilization to implantation, the embryo depends on the function of existing mitochondria [10,11,12].

The mitochondrial genome alterations involved in the ovarian aging process are both qualitative and quantitative [4]. The former include mtDNA damage caused by oxidative stress, the accumulation of acquired mtDNA mutations, the effects of inherited mtDNA mutations, and alterations in the mitochondrial stress response mechanism [4,13]. The latter refer to alterations in the oocyte mtDNA copy number. Notably, there are also multiple dysfunctions in mitochondrial signaling pathways involved in the process of cellular aging. These include alterations in mitochondrial fusion and fission, defective mitochondrial electron transport chain (ETC), and dysfunctions in mitochondrial metabolism and membrane potential [13]. The quantitative hypothesis was also tested by investigating the impact of mtDNA content alterations in granulosa cells (GCs), embryonic cells and peripheral blood cells on fertility. In vitro fertilization (IVF) represents an excellent study model to investigate these issues.

The present review aims to synthesize the evidence about: (1) the effect of qualitative and quantitative mtDNA alterations on female fertility, paying particular attention to those with a pathophysiology characterized by a relevant role of oxidative stress; (2) the use of oocytes, GCs, embryonic cells and peripheral blood cells mtDNA content as a female fertility surrogate biomarker; (3) experimental therapies tested to try to subvert the ovarian aging process with particular reference to antioxidant treatments.

Pubmed, Medline, Embase, and Scopus were systematically searched from their inception to 1 December 2020. Two separate searches were conducted using the following terms: (1) “mitochondrial DNA” OR “mtDNA” OR “mitochondrial DNA copy number” OR “mitochondrial DNA content” AND “oocyte” OR “granulosa cell” OR “embryo” OR “blood cell”; (2) “ovarian aging” OR “fertility” AND “oxidative stress” OR “antioxidants”. After a full review of titles, abstracts and, in case of doubt, of full texts, a total of 65 studies were included in the qualitative synthesis.

## 2. Qualitative mtDNA Alterations

### 2.1. Oxidative Stress in Mitochondrial DNA-Dependant Aging

Mitochondrial dysfunction has been shown to have a pivotal role in the aging process [14]. In this regard, the effect of oxidative stress on mtDNA emerged as a factor of considerable importance. The hypothesized mechanism involves the damage induced by the reactive oxygen species (ROS) produced by mitochondria on mtDNA which can compromise the integrity of the respiratory chain and, as a consequence, fuel the mitochondria-dependent aging process [14].

One of the cornerstones on which ROS-induced mutagenicity is founded is the modification of the DNA bases. In particular, it has been shown that the somatic mtDNA of older subjects is characterized by a strong G > A transition mutation preference [14,15,16]. An alternative theory proposed to justify the high incidence of mtDNA point mutation typical of the aging process was the limitation in fidelity of mitochondrial polymerase γ (POLG) principally due to the decreased exonuclease proofreading activity [14,17,18,19]. Finally, the formation of mtDNA double-strand breaks induced by ROS seems to be involved in the somatic mtDNA deletions generation [14].

According to the most accepted theory, mtDNA mutations would cause a loss of synchrony in functional ETC proteins production by mtDNA and nuclear DNA (nDNA) leading to partial uncoupling of the respiratory chain. As a consequence, due to the lack of protective histones and considering the close proximity of mtDNA to ETC, the mtDNA mutation rate significantly grows. This result in a vicious circle: the mtDNA mutations would cause further accumulation of ROS, and an exponential increase in mtDNA mutations and ATP reduction [13].

Importantly, although extensively studied, some of these mechanisms are more complex and only partially elucidated. In fact, the mitochondrial structure is formed not only by the mtDNA but also by a great number of nucleoid associated proteins which play a relevant role in maintenance of mtDNA and gene expression. Oxidative stress may determine significant nucleoids structural modifications leading to a deterioration of their dynamics and to a severe mitochondrial dysfunction [20]. Furthermore, also the association between high levels of mtDNA mutations and ROS production is still under debate. Indeed, Trifunovic et al. observed that in mice expressing an error-prone version of the catalytic subunit of mtDNA polymerase, the accumulation of mtDNA mutations was not associated with an increased ROS formation. They proposed that the respiratory chain dysfunction per sé might be the primary inducer of the aging process in mtDNA mutator mice [21].

Finally, the source of ROS deemed responsible for the mtDNA damage has yet to be fully clarified. In this regard, the ROS generation by inflammation-linked processes needs future investigation [14].

### 2.2. Oxidative Stress in Ovarian Follicle Mitochondrial DNA-Dependant Aging

Oxidative stress has an inherent role in many mechanisms involved in the process of ovarian aging. Here, we exclusively focused on its role in the context of mtDNA dependent aging. A Graffian follicle consists of an oocyte, an antrum filled with the follicular fluid, and the mural GCs (mGCs) lining the wall of the follicle. The cells that surround the oocyte, and project into the antrum are called cumulus granulosa cells (CGCs). Oocytes secrete factors that can regulate the GCs functions and GCs form the follicular microenvironment for oocyte developmental competence [22,23,24]. Mitochondrial dysfunction and mtDNA alterations in all the aforementioned cells have been proposed as key elements in the ovarian aging and infertility pathophysiology. Indirect evidence also suggests a pivotal role of mitochondrial POLG in qualitative ovarian depletion. In fact, in a well conducted and interesting contribution, Louma et al. showed that parkinsonism and premature menopause cosegregate with POLG defects. These findings also lay the foundations for future investigations. In fact, according to authors, the progressive external ophthalmoplegia associated to POLG mutations can be considered as a source of information to understand the role of mitochondrial dysfunction and oxidative stress in the etiopathogenetic process of both Parkinson’s disease and premature menopause [25].

### 2.3. Evidence about mtDNA Oocyte Alterations

**Figure 1:**
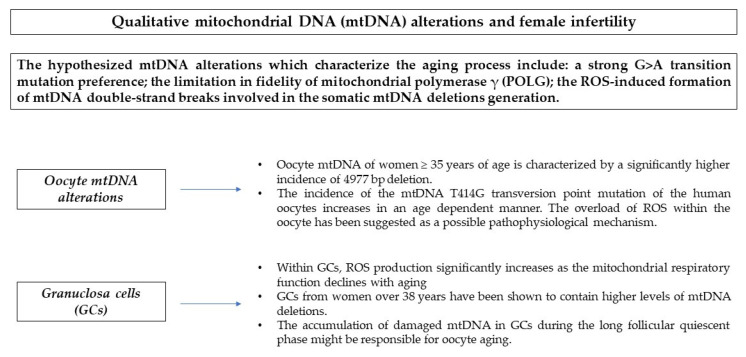
Qualitative mtDNA alterations and female fertility.

Important contributions suggest that oocytes mtDNA mutations and deletions could have a role in the age-related decline of female fertility. As proof of this, Chan et al. investigated the mtDNA content and the incidence of 4977 bp deletion in unfertilized MII oocytes. Notably, this deletion is considered one of the most frequent mtDNA mutations, accumulates with advancing age in various tissues and, according to some authors, can also be considered a marker of natural aging [26]. The results showed that its incidence was equal to 34.6%. Furthermore, they observed that women ≥ 35 years of age were characterized by a higher incidence of 4977 bp deletion, a lower mtDNA copy number, a higher follicle stimulating hormone (FSH) serum concentration, and a poorer ovarian response when compared with younger women [26]. Barritt et al. investigated the presence of a point mutation (T414G) in the mtDNA control region of oocytes from women of advanced age. They analyzed 66 non-viable discarded human oocytes and observed that in the younger group of patients (26–36 years old women) this mtDNA point mutation was present in only 4.4% of oocytes compared to 39.5% from the older group (37–42 years old women) (*p* < 0.01) [27] (Figure 1). Interestingly, they concluded that the incidence of the mtDNA T414G transversion point mutation of the human oocytes increases in an age dependent manner. The potential clinical application of this point mutation might be its association with ovarian and reproductive aging. Furthermore, considering that this mutation affects the mtDNA control region, it has been hypothesized that the regulation of mtDNA transcription and replication could be altered during the oocyte and embryo development [27]. The overload of ROS within the oocyte has been suggested as a possible pathophysiological mechanism. However, there is still lack of robust data to support this hypothesis.

### 2.4. Evidence about mtDNA Granulosa Cell Alterations

The maintenance of an energetic homeostasis in the ovary and, in particular, in the follicles enables the development of a competent oocyte. The process of oocyte aging is thus probably related to that of the whole ovary. In this regard, recent findings tend to confirm the key role of oxidative stress in aged GCs [28]. Within GCs, ROS production significantly increases as the mitochondrial respiratory function declines with aging [2]. At the same time, a reduction in coenzyme Q-dependent mitochondrial respiratory chain activity in mGCs has been observed [29]. Not surprisingly, GCs from women older than 38 years have been reported to contain higher levels of mtDNA deletions [30] and damaged mitochondria [31] (Figure 1). The pathophysiological mechanisms behind these mitochondrial homeostasis perturbations are still under investigation. Recent data indicate a reduced expression of the peroxisome proliferator-activated receptor gamma coactivator 1-alpha (PPARGC1A) gene in the CGCs of women with diminished ovarian reserve (DOR) compared to those with normal ovarian reserve (NOR) [32]. The product of this gene is involved in mitochondrial biogenesis and respiration [33] and in protection against ROS related damage. In a goat experimental model, this gene has been shown to play a role in follicular development [2,34]. The long process of folliculogenesis and the network between GCs, oocytes, and the ovarian microenvironment are the key factors that allows the oxidative stress process to spread and determine the qualitative detrimental effect on ovarian reserve. Accordingly, the accumulation of damaged mtDNA in GCs during the long follicular quiescent phase might be responsible for oocyte aging [2,28]. In other words, in older women, the GCs in primordial follicles and, as a consequence, the oocytes have been under the effect of low amounts of ROS derived from the process of mitochondrial respiration for a long time, and thus probably suffered damage to their mitochondria and mtDNA [31]. At the same time, the ovarian microenvironment is altered during aging and characterized by a ROS overload [2,35,36]. Importantly, ROS have a detrimental effect only at supraphysiologic levels. In fact, a good balance between ROS and antioxidant activity ensures a successful development and maturation of the oocyte [37]. Another aspect that still deserves to be clarified is the possible hermetic effect of ROS on ovarian cells. A deeper knowledge of this mechanism and the identification of the specific form of ROS that triggers hormesis could be of great help in understanding the reproductive aging process [38].

Interestingly, Lin et al., in an experimental model, induced GCs senescence with a 24 h hydrogen peroxide treatment. At the end of the exposure, the analysis of the mitochondrial membrane potential (MMP) revealed that the exposure to H_2_O_2_ determined a considerable decrease in the depolarization potential of membranes, suggesting a possible damage to mitochondria in the aged GCs. The total cellular and mitochondrial ROS of the H_2_O_2_-treated group was higher when compared to that of the control group. Authors also observed a significant reduction in the mtDNA copy number in senescent cells characterized by a lack of mitochondrial sufficiency [39].

In a recent contribution, Liu et al. proposed the study of mGCs taken from patients undergoing assisted reproductive technology (ART) as a good model for investigating the age-related ovarian altered function. The mGC microenvironment is in fact very similar to that of oocytes. The authors studied the mitochondria in mGCs and found age-related changes of mitochondrial ultrastructure, accompanied by decreased MMP in the older group of patients. However, they also failed to observe a significant decline of intracellular ROS leves, mtDNA content and integrity with aging. Finally, the declined ATP production and the reduced OXPHOS function were hypothesized to be responsible for age-related dysfunction of mGCs [24].

### 2.5. Mitochondrial Unfolded Protein Response (mtUPR)

The mitochondrial unfolded protein response (mtUPR) is a stress response pathway characterized by either a depletion of the genome of mitochondria or the accumulation of misfolded proteins within these organelles [40,41]. According to the most recent evidence, mtUPR regulates a large set of genes involved in protein folding, ROS defenses changing, metabolism and innate immune response modulation [42,43].

This pathway was first studied in *Caenorhabditis elegans*. Interestingly, mitochondrial matrix caseinolytic peptidase (CLPP), a pivotal regulator of mtUPR, was observed to be upregulated after stress in mitochondria induced by unfolded proteins [40,44]. Importantly, mtUPR is also involved in coenzyme Q biosynthesis, glycolysis, and mitochondrial fission [1]. On the contrary, impaired mtUPR promotes the accumulation of damaged proteins in an age dependent manner, reduces OXPHOS processes and increases ROS production. These mechanisms emerged as potentially important within the process of reproduction as the global germline deletion of CLPP would be able to determine infertility and accelerated follicular depletion. In this regard, the partial rescue of fertility phenotype in CLPP-deficient mice produced with the administration of drugs that inhibit the target of rapamycin (mTOR) further sustains this hypothesis. Well conducted studies are warranted in order to test this pathway as a new treatment goal for the promotion of fertility during the aging process [45].

## 3. Quantitative Alterations

MtDNA copy number is considered a quantitative measure of the mtDNA cell content. This biomarker attracted the attention of reproductive medicine researchers. In particular, the association between its quantification in oocytes, GCs, embryonic cells, and peripheral blood cells with surrogate fertility outcomes such as the embryo implantation and pregnancy rates has been extensively investigated (In this scenario, IVF emerged as the ideal study model.

### 3.1. Oocytes mtDNA Content and Fertility

The initial number of mitochondria and the mtDNA content of the oocyte emerged as key factors in the process of fertilization and embryonic development in different mammalian species [2]. In a pioneering study, Reyner et al. observed that the average mtDNA copy number in human oocytes was significantly lower in cohorts of patients suffering from fertilization failure compared to those with a normal fertilization rate [8]. They concluded that low mtDNA oocyte content might be the consequence of inadequate mitochondrial biogenesis or cytoplasmic maturation and might adversely affect the oocyte fertilizability [8]. Some years later, Santos et al. confirmed these findings and reaffirmed that mtDNA oocyte content should be considered as critical to fertilization outcome and that it might serve as an important marker of oocyte quality [46]. In particular, the authors compared the mtDNA content of 35 fertilized oocytes with that of 65 unfertilized oocytes and observed a significant higher mtDNA copy number in the first group [46]. Not surprisingly, low mtDNA content is associated with the impaired oocyte quality observed in women with ovarian insufficiency [8]. Furthermore, polar bodies from oocytes of women at an advanced reproductive age have been shown to have less mtDNA than those of younger women [47,48]. These findings partially explain the pivotal role of mtDNA oocyte content in the reproductive success. In fact, the amount of mitochondria of the fertilizable oocyte must be sufficient for its distribution among the embryonic blastomeres in order to guarantee the optimal functioning of each cell until the resumption of mtDNA replication [2].

### 3.2. Granulosa Cells mtDNA Content and Fertility

**Figure 2:**
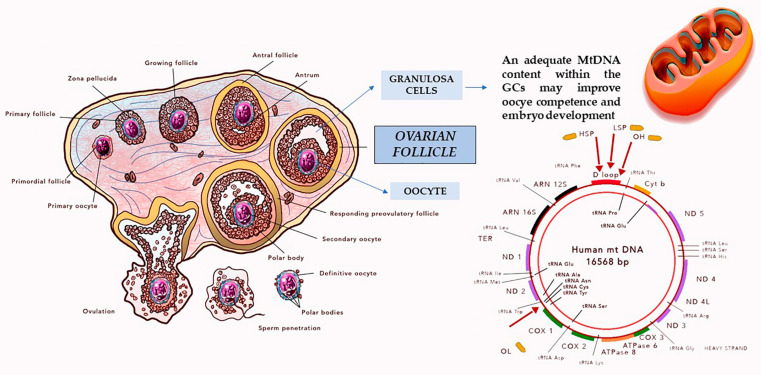
The oocyte competence is influenced by the communication between the oocyte and the surrounding granulosa cells (GCs). GCs express factors implicated in mtDNA replication and maintenance and therefore play a notable role in creating an adequate mtDNA pool during oogenesis.

Within the ovarian follicle, oocyte competence is determined by a network between the oocyte and the surrounding GCs [23]. The study of oocyte often implies its destruction. In this context, CGCs emerged as one of the best non-invasive experimental models for investigating the metabolic process conditioning the oocyte quality and the embryo development potential [49]. Boucret et al. showed that CGCs, through the expression of factors involved in the replication and maintenance of mtDNA, play a pivotal role in the constitution of a sufficiently large mtDNA pool during oogenesis (Figure 2) [32]. From this, evidence emerges that the mtDNA content within the CGCs may contribute to the oocyte competence and supporting normal embryo development [23]. In order to confirm this hypothesis, Desquiret-Dumas et al. conducted an interesting study aimed at establishing if mtDNA content of CGCs is related to oocyte competence. Noteworthy, they did not find an association between CGCs mtDNA content and oocyte maturity or fertilizability. On the other hand, a significant link was observed between the CGCs mtDNA content and the embryo quality. In particular, a significantly higher mtDNA content was associated with a good quality embryo development. Authors concluded suggesting the quantification of CGCs mtDNA copy number as a possible new biomarker of embryo viability. However, the individual characteristics make it very difficult to establish a general threshold value which can be considered as valid for all patients [23]. Taugourdeau et al. confirmed these findings and showed a significantly increased mtDNA copy number in CGCs of embryos that successfully implanted when compared with that of non-implanted embryos. Importantly, multivariate analysis, taking into account the age of included women, the quality of the embryos, and the Antimüllerian hormone (AMH) serum concentration, suggested an independent relationship between the mtDNA copy number in CGCs and the embryo implantation capacity [50].

More recently, Lan et al. focused on the association between the mtDNA copy number of CGCs and the maturation of oocyte cytoplasm. Results showed that the mtDNA content of CGCs decreases from the germinal vesicle (GV) phase to the metaphase I (MI) phase and it does not undergo changes from the MI to MII stage [51]. At different oocyte maturation stages, the CGCs mtDNA may self-degrade and replicate to meet the energy requirements of the corresponding oocyte [49,51].

### 3.3. Embryonic Cells mtDNA Content and Fertility

Mature oocytes of women who undergo IVF cannot be invasively analyzed once they are fertilized. Not surprisingly, researchers shifted their attention to search a correlation between mtDNA content of human embryonic cells and reproductive outcomes [52]. Noteworthy, the mtDNA copy number in embryos represents the mtDNA content of oocytes before fertilization because the mtDNA replication process stops until the blastocyst stage [10].

Different authors have examined the association between human blastocyst mtDNA content, female age, embryo chromosome status, viability, and implantation potential [9,53,54,55,56,57,58,59,60,61,62,63]. For the sake of clarity, we reported their results in Table 1.

**Figure 3:**
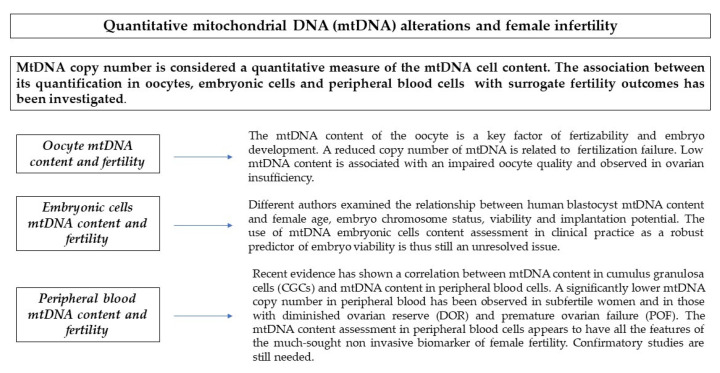
Quantitative mtDNA alterations and female fertility.

Unfortunately, their findings are conflicting. The use of mtDNA embryonic cells content assessment in clinical practice as a reliable predictor of embryo viability is thus still a debated issue [51,52] (Figure 3). Different research methods can significantly affect the predictive power of mtDNA copy number [63]. In particular, MtDNA quantification method should be probably standardized before proceeding with research in this field. Wells et al. strongly recommend that investigators should avoid the use of old and/or poorly stored samples, never normalize mitochondrial data against a single copy nuclear DNA sequence, and exercise caution when using Whole Genome Amplification (WGA) methods that provide a non-uniform coverage of the mitochondrial genome or Next Generation Sequencing (NGS) techniques that produce relatively few mtDNA reads [64].

Well conducted contributions focusing on mGCs, CGCs, embryo culture medium, and trophectoderm biopsies are thus warranted. With the use of appropriate methods and collaborative efforts, this still unclear issue of preimplantation embryo biology, and its implication for clinical practice might soon be better understood [63,64].

### 3.4. Peripheral Blood Cells mtDNA Content and Fertility

Of utmost interest is the recent evidence demonstrating an association between the mtDNA copy number in CGCs and that assessed in peripheral blood cells [65,66,67]. Measuring blood mtDNA content is simple, rapid, and minimally invasive. Its use in the development of diagnostic tool for ovarian aging thus emerged as an intriguing issue [66]. In order to shed light on this peripheral biomarker, our group conducted a nested case–control study. Women were recruited at the time of screening for aneuploidies in the first trimester of pregnancy. The mtDNA peripheral blood cells content of women that were seeking pregnancy for more than 12 months (subfertile women) was compared with that of controls of the same age who achieved pregnancy in less than 12 months (fertile women) [67]. Interestingly, we found a significantly decreased mtDNA content in blood cells of subfertile women. From the ROC curve analysis emerged that participants with a mtDNA/nDNA below 105 had a more than fivefold higher risk of being subfertile. Interestingly, the discriminatory ability of this biomarker was shown to be increased in younger women [67].

In a previous contribution, Bonomi et al. found a diminished mtDNA copy number in peripheral blood cells of women with DOR. A biological grading showing the lowest levels in subjects with premature ovarian failure (POF) and the highest in those with a normal ovarian reserve was also observed. Noteworthy, an intermediate level of mtDNA/nDNA copy number was reported in patients with a poor response to controlled ovarian hyperstimulation (COH) for IVF [65]. Further independent studies are warranted to test the association between mtDNA/nDNA copy number in peripheral blood and premature menopause time of onset. In fact, one could speculate that blood cell mtDNA content assessment might constitute a reliable predictive test in the occult phase of the disease, when the available biomarkers have a still insufficient efficiency [65].

The interpretation of these findings is difficult. The low mtDNA content assessed in blood cells could be a peripheral indicator of a low mtDNA copy number in the oocytes which would cause a fertilization impairment and, as a consequence, a lower reproductive capacity. This hypothesis is fascinating. However, the evidence demonstrating an association between mtDNA copy number in oocytes or follicular cells and mtDNA content in peripheral blood cells is still weak [67].

Alternative explanations may also be valid. One may hypothesize that oxidative stress, per sè, acts as the real mediator of fertiliy damage and that the reduction in peripheral blood mtDNA copy number is only the reflection of a high ROS level [68]. Notably, if compared to its nuclear genome, mtDNA accumulates damage more extensively when exposed to ROS [69]. Original data reporting a relationship between the ROS amount and the mtDNA content in peripheral blood are lacking. However, published evidence shows a reduced peripheral blood cells mtDNA copy number in subjects affected by conditions traditionally characterized by an excessive oxidative stress such as metabolic syndrome and coronary heart disease [70,71,72]. Investigators should consider this aspect in their future contributions and try to quantify in peripheral blood the oxidative stress markers level along with the mtDNA copy number in fertile and subfertile women.

The study of the mtDNA copy number in peripheral blood deserves considerable attention. If these preliminary results were confirmed, this biomarker could constitute a particularly important tool in the clinical setting. In fact, to date, the commonly used ovarian reserve markers (i.e., AMH serum concentration, basal FSH serum concentration and antral follicle count (AFC)) have been shown to be reliable in predicting the number of follicles and oocytes that a woman possesses at a particular time in her life but extremely weak in reflecting the quality of gametes and hence the fecundity. The assessment of mtDNA copy number in peripheral blood seems to have all the characteristics to fill this gap and to become the much-sought noninvasive biomarker of female fertility [68] (Figure 3).

## 4. Proposed Interventions

Several research efforts have been done with the aim of reducing or reversing the detrimental impact of mitochondrial alterations on reproduction and thus improving the energy production in gametes and embryos [1].

The principal proposed strategies include autologous mitochondria transfer, heterologous mitochondria transfer and administration of bioactive molecules, which protect against oxidative damage and improve the overall capacity of energy production. Available evidence about mitochondria transfer has been excellently summarized in previous contributions [1,10,48] and its synthesis is beyond the scope of this review. Herein, we focused on evidence about proposed bioactive nutrients for optimizing mitochondrial function tested in animal or human models.

Coenzyme Q10 (CoQ10) is a very important electron carrier in the mitochondrial ETC [72], has critical antioxidant properties, controls cellular redox, influences transcriptional activity of cells and is required for the activity of succinate dehydrogenase [29]. Recent evidence demonstrates that CoQ10 supplementation in an aged animal model delays depletion of ovarian reserve, restores oocyte mitochondrial gene expression, and improves mitochondrial activity [29,73]. Interestingly, Boots et al. also demonstrated that CoQ10 supplementation to mice fed with high-fat/high-sugar (HF/HS) diet improves several aspects of oocyte quality but does not completely prevent the effects of obesity [74]. Bentov et al. conducted a double-masked randomized placebo-controlled trial that included patients between 35 and 43 years of age selected for IVF [75]. The included subjects were treated with either 600 mg of CoQ10 or placebo. They compared the post-meiotic rate of aneuploidy using polar body biopsy and comparative genomic hybridization. Unfortunately, owing to the premature termination of the study for safety reasons, authors failed to reach the number of participants defined by the power calculation. Published data showed a lower rate of aneuploidy in the group treated with CoQ10. However, the observed outcome differences did not achieve statistical significance [76].

Resveratrol is a potential antiaging polyphenolic compound from plants and is mainly present in red wine. Evidence shows that resveratrol enhances the activity of Sirt1, inhibits phosphodiesterase, and has a beneficial effect on mitochondrial activity [76,77]. Importantly, in vitro and in vivo studies demonstrated a variety of biologic properties including antioxidant, anti-inflammatory, anticarcinogenic, and antiproliferative effects [77]. Recently, Liu et al. designed a study aimed at evaluating the impact of resveratrol on oocyte maturation in aged mice and humans. They studied in vitro culture in the presence of three different resveratrol concentrations (0.1, 1.0, and 10 µm) or dimethyl sulfoxide. The authors observed that resveratrol at a concentration equal to 1.0 µm significantly increased the emission rate of first polar body in oocytes of aged mice and humans, and the chances of fertilization and blastocyst formation in aged mice. Moreover, the immunofluorescence intensity of mitochondria and normal morphology of spindle and chromosome of oocytes undergoing in vitro maturation (IVM) were markedly improved compared with control samples in aged mice and humans [76]. Resveratrol may be considered promising in promoting mitochondrial function in human oocytes. However, its exact therapeutic use and safety remain to be determined [4].

In order to shed light on the ability of oral antioxidants supplementation to improve fertility outcomes, Showell et al. recently conducted a Cochrane meta-analysis [78]. Investigators compared oral antioxidants (i.e., combinations of antioxidants, *N*-acetylcysteine, melatonin, L-arginine, myo-inositol, carnitine, selenium, vitamin E, vitamin B complex, vitamin C, vitamin D + calcium, CoQ10, and omega-3-polyunsaturated fatty acids) with placebo, no treatment or standard treatment or another antioxidant. Considering the very low-quality of the evidence, they were uncertain whether antioxidants improve live birth rate compared with placebo or no treatment or standard treatment (odds ratio (OR) 1.81, 95% confidence interval (CI) 1.36 to 2.43; *p* < 0.001, I^2^ = 29%; 13 randomized clinical trials (RCTs)). After the pooling of results, low-quality evidence showed that a lower dose of melatonin was not associated with an increased live-birth rate when compared with a higher dose (OR 0.94, 95% CI 0.41 to 2.15; *p* = 0.89, I2 = 0%; 2 RCTs). The authors concluded that there is low- to very low-quality evidence in order to support a benefit of antioxidants on female subfertility [78].

Further randomized placebo-controlled trials providing evidence of good quality are needed to assess benefits or harms or both of supplemental antioxidants for subfertile women. Robust and convincing evidence is thus warranted before considering the introduction of antioxidants supplementation in clinical practice.

## 5. Conclusions

The impact of oxidative stress on ovarian follicle cells mtDNA has an inherent role in many mechanisms involved in the process of ovarian aging. The effect of mtDNA alterations in GCs on qualitative ovarian reserve depletion is supported by the most robust evidence. Considering their strict network, the GCs mtDNA alterations are probably similar to those in their follicular cells. However, direct evidence about the impact of ROS on oocytes mitochondrial genome are scanty. Future studies should thus focus on the impact of oxidative stress on oocyte mtDNA qualitative alterations and on its effect on oocyte competence.

In recent years, the mtDNA embryonic cells content emerged as a possible measure to determine the embryo implantation potential. Unfortunately, published results are conflicting in every aspect and hopefully results from ongoing RCTs will shed some light on this issue.

MtDNA content assessment in peripheral blood seems to have all the characteristics of the much-sought non-invasive biomarker of female fertility [67]. However, further well conducted prospective studies are needed before considering its introduction into clinical practice.

In more general terms, the strength of the evidence provided by the present review is limited by the absence of a quantitative synthesis. Unfortunately, this was not possible due to the different study designs and methods adopted by the different authors. The quantitative and qualitative mtDNA variations observed in ovarian, embryonic, and peripheral blood cells represent promising tools to predict female fertility. However, in order to confer them a clinical significance, future research initiatives should strive to establish the association between these alterations and quantitative reproductive outcomes (i.e., fertilization, pregnancy, and live birth rates) using experimental methods as standardized and reproducible as possible. Here again, responses to controlled ovarian hyperstimulation (COH) and IVF treatments constitute the ideal surrogate markers of female fertility to study in order to untangle this issue.

## Figures and Tables

**Table 1:** Characteristics of studies investigating the association between mtDNA embryonic cells content and fertility outcomes.

Study	Design	Biopsy Site	Nr. of Embryos/Blastocysts Analyzed	mtDNA Quantification Method	Impact of mtDNA Copy Number on Implantation	Association between mtDNA Copy Number and Female Age	Impact of mtDNA Copy Number on Euploidy	Impact of mtDNA Copy Number on Embryo Morphology
Fragouli et al., 2015 [9]	Prospective study	Trophectoderm	379 embryos	Relative quantification	Euploid blastocysts that successfully implanted were shown to contain a lower mtDNA quantity than those failing to implant	Significantly higher quantity of mtDNA in embryos from older women	mtDNA levels were elevated in aneuploid embryos	NR
Diez-Juan et al., 2015 [53]	Retrospective study	Trophectoderm	310 euploid embryos	Relative quantification	The relative mtDNA content of day-3 and euploid embryos that successfully implanted resulted significantly lower than that of those that failed to implant	The quantity of mtDNA was significantly higher in embryos from older women	NR	Trend toward an increased mtDNA content in poorer quality embryos
Spinella et al., 2016 [54]	NR	Trophectoderm	96 blastocysts	Relative quantification	Blastocysts that implanted and resulted in baby born, were shown to contain lower mtDNA quantities compared with those that failed to implant	NR	The relative quantity of mtDNA was significantly lower in euploid embryos	Fully expanded (Grade 5 or 6) euploid blastocysts had an mtDNA average value 1.6-fold lower than euploid blastocysts with expansion grade 3
Ravichandran et al., 2017 [55]	Retrospective study	Trophectoderm	1505 euploid blastocysts	Relative quantification	Of embryos containing ‘elevated’ amounts of mtDNA, none implanted	Female patients in the youngest age bracket had average mtDNA levels that were significantly lower than those in the oldest age bracket	NR	None
Victor et al., 2017 [56]	Retrospective study	Trophectoderm	1396 embryos	Relative quantification	Absence of an association	No differences in mtDNA scores between age groups	Absence of an association	NR
Fragouli et al., 2017 [57]	Prospective study	Trophectoderm	199 euploid blastocysts	Relative quantification	Elevated mtDNA was accompanied by implantation failure	The mtDNA quantity appeared to increase with advancing female age, although the difference was not statistically significant	NR	NR
Treff et al., 2017 [58]	Retrospective study	Trophectoderm	374 euploid blastocysts	Relative quantification	None	mtDNA copy number was negatively correlated with oocyte age	NR	NR
de Los Santos et al., 2018 [59]	Retrospective study	Trophectoderm	1641 biopsied blastocysts	Relative quantification	NR	Neither age nor AMH levels were found to be associated with mtDNA quantity	Lower amount of mtDNA in euploid blastocyst	Blastocysts with poor quality TE had more chances of carrying higher mtDNA values
Lledo et al., 2018 [60]	Prospective study	Trophectoderm	159 blastocysts	Relative quantification	Reduction in ongoing pregnancy rate associated with elevated mtDNA copy number	mtDNA copy number was negatively correlated with female age	NR	NR
Klimczak et al., 2018 [61]	Retrospective study	Trophectoderm	1510 blastocysts	Relative quantification	None	No correlation between mtDNA content and the patients’ age	NR	Embryos with higher mtDNA content were found to be of poorer quality (grade 3) relative to grades 1 and 2
Lee et al., 2019 [62]	Double-blind, observational, prospective	Blastomere or Trophoectoderm	1617 embryos	Realative quantification and adjusted calculation	Absence of an association	Significant but weak correlation	Adjusted mtDNA quantification significantly lower in euploid blastocyst	NR
Scott et al., 2020 [63]	Prospective study	Trophectoderm	615 euploid blastocysts	Relative quantification	Absence of an association	No correlation was observed between maternal age and relative mtDNA copy number	NA	NR
